# T Cell Membrane Mimicking Nanoparticles with Bioorthogonal Targeting and Immune Recognition for Enhanced Photothermal Therapy

**DOI:** 10.1002/advs.201900251

**Published:** 2019-06-11

**Authors:** Yutong Han, Hong Pan, Wenjun Li, Ze Chen, Aiqing Ma, Ting Yin, Ruijing Liang, Fuming Chen, Yifan Ma, Yan Jin, Mingbin Zheng, Baohong Li, Lintao Cai

**Affiliations:** ^1^ Guangdong Key Laboratory of Nanomedicine CAS‐HK Joint Lab of Biomaterials Shenzhen Engineering Laboratory of Nanomedicine and Nanoformulations Shenzhen Institutes of Advanced Technology (SIAT) Chinese Academy of Sciences Shenzhen 518055 P. R. China; ^2^ Dongguan Key Laboratory of Drug Design and Formulation Technology Key Laboratory for Nanomedicine Guangdong Medical University Dongguan 523808 P. R. China

**Keywords:** biomimetic nanoparticles, bioorthogonal chemistry, photothermal therapy, T cell membranes, tumor dual targeting

## Abstract

Due to specific immune recognition receptors on the surface of T cells, their membranes are promising mimic nanocarriers for delivering drugs to tumor lesions. However, this single targeting strategy potentially compromises therapy efficacy for tumor targeting due to inter‐ and intra‐heterogeneity of tumors. Azide (N_3_) or bicyclo [6.1.0] nonyne (BCN) modified unnatural sugars can be successfully incorporated into surface glycans of various tumor cells as artificial receptors, which is expected to overcome the insufficiency of single targeting. Based on this artificial tumor targeting strategy, indocyanine green nanoparticles (INPs) coated with N_3_‐labeled T cell membrane (N_3_‐TINPs) are constructed, which can specifically target the natural antigen and BCN artificial receptors on tumors through immune recognition and bioorthogonal chemistry, respectively. The results show that the fluorescence intensity in the tumors of mice treated with N_3_‐TINPs is 1.5 fold compared with that of the mice treated with unlabeled TINPs. The accumulated N_3_‐TINPs in the tumor significantly increase the photothermal therapeutic effect without adverse effect. Therefore, this T cell membrane mimicking nanoparticles based bioorthogonal chemistry may provide an alternative artificial targeting strategy for further tumor targeting photothermal therapy.

## 1 Introduction

As a promising strategy of cancer therapy, photothermal therapy (PTT) shows many advantages such as high efficacy, noninvasive, and noninjury to normal tissues.^1^ The PTT capitalize the photothermal conversion performance of nanomaterials, can convert optical energy into thermal energy to eliminate tumor effectively.^2^ However, PTT mediated by nanomaterials is limited by the insufficient accumulation of nanoparticles in tumor site. To enhance the PTT effect, targeting ligands modification or by using cell membrane coated technique could be utilized to increase the drug accumulation in tumor.^3^ Cell membrane coated nanoparticles have emerged as a promising strategy for tumor‐targeting, due to the retain of complex natural properties of the source cells.^4^ Compared with other cells, immune cells including T cells have unique site‐specific targeting properties,[qv: 3c,5] which can be used for tumor targeting.^6^ Due to the specific immune recognition proteins on T cells membrane, e.g., T cell receptors (TCRs), the activated T cells can recognize correlated molecules on tumor surface,^7^ exhibiting a natural and high tumor affinity. Take advantage of the immunological recognition properties of T cells, their membrane would be a promising carrier that targeting tumor for drug delivery. However, single targeting strategy is insufficient due to inter‐ and intra‐heterogeneity of tumors, dual‐targeting strategy may provide a more promising method to enhance the accumulation of nanoparticles in malignant lesion.^8^

Recently, an artificial targeting strategy based on bioorthogonal metabolic glycoengineering has been widely reported.^9^ Metabolic glycoengineering is a powerful technique that introduces various chemical groups to cellular glycan with the treatment of unnatural monosaccharide.^10^ Particularly, this technique can artificially generate bioorthogonal groups on the tumor as artificial “receptor‐like” target, using for selective targeting in complex environments.^11^ Based on this, our group reported a novel bicyclo [6.1.0] nonyne (BCN) modified unnatural sugars (Ac_4_ManN‐BCN), which can efficiently and nondestructively be incorporated into wild tumor cell surface glycans.^12^ The BCN motif on the cell surface act as an excellent targeting tag and effectively enhanced the tumor recognition of T cells modified with azide (N_3_) though bioorthogonal click chemistry.

**Figure 1:**
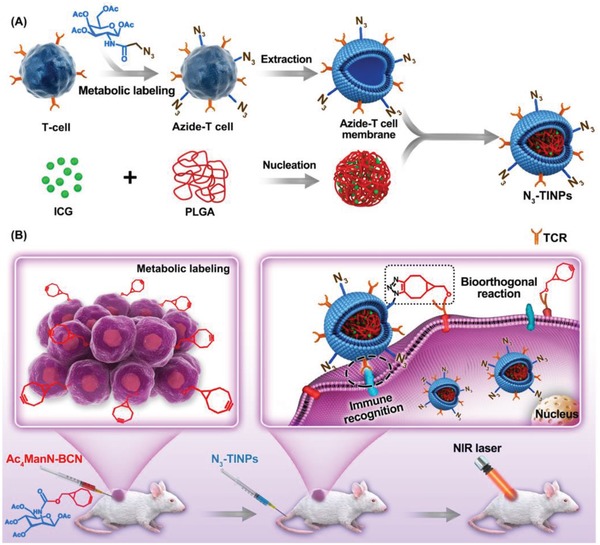
Schematic illustration of N_3_‐labeled T cell membrane‐biomimetic nanoparticles with dual‐targeting mechanism for highly efficient photothermal therapy. A) Synthesis of N_3_‐TINPs. Extracting N_3_‐labeling T cell membranes were coated on prepared ICG‐PLGA polymeric cores by extrusion to form N_3_‐TINPs. B) Tumor cells carrying the BCN group via natural glycometabolic labeling by pretreatment with Ac_4_ManN‐BCN. N_3_‐TINPs could target tumor through immune recognition of T cell membrane and bioorthogonal reaction between BCN and N_3_ groups, and effectively eliminate mouse tumors through ICG‐mediated photothermal effects.

In this study, we extract an N_3_‐labeled T cell membrane to coat nano‐photosensitizer (indocyanine green, ICG) for increasing its effective accumulation in tumor and promote its photothermal therapy. As shown in **Figure** **1**, the N_3_ group was labeled on the activated T cell membrane via glycometabolism by pretreatment with the azido sugar Ac_4_GalNAz. Tumor cells carried the BCN group via natural glycometabolism by the previously intratumorally administered BCN‐sugar Ac_4_ManN‐BCN. ICG, as photosensitizer, was loaded into poly (lactic‐*co*‐glycolic acid) (PLGA) to form an ICG‐PLGA polymeric core and then coated with the extracted N_3_‐labeled T cell membrane, resulting in N_3_‐labeled T cell membrane‐coated ICG‐PLGA nanoparticles (N_3_‐TINPs). By translocating T cell membranes to the nanoparticles, the biological targeting properties of the T cells can be conferred to N_3_‐TINPs as well, allowing them to target tumor through the retained immune recognition receptors. Further, the bioorthogonal reaction between the N_3_ and BCN groups enhanced the anchoring of the N_3_‐TINPs in tumor. The dual‐targeting strategy facilitated the accumulation of nano‐photosensitizer, and the accumulated N_3_‐TINPs efficiently eradicated tumors through ICG‐mediated photothermal effect. Overall, our study provides a nondestructive targeting modification strategy, showing that N_3_‐TINPs can act as a new delivery platform for effective drug accumulation and ultimately achieve highly efficient PTT effect.

## 2 Results and Discussion

### 2.1 Preparation and Characterization of N_3_‐TINPs

**Figure 2:**
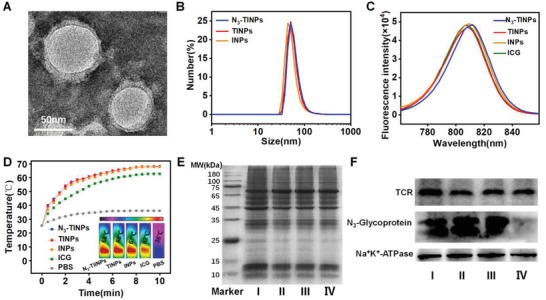
Characterization of dual‐targeting N_3_‐TINPs. A) TEM image of N_3_‐TINPs. B) Size distribution of N_3_‐TINPs, TINPs, and INPs. C) Fluorescence spectra of different nanoparticles and free ICG. D) Infrared thermal images and temperature curve. E) SDS‐PAGE protein analysis. F) Western blotting analysis of TCR and N_3_‐glycoprotein. I: T cell lysate, II: T cell membrane vesicle, III: N_3_‐TINPs, IV: TINPs.

According to procedure in the literature,^13^ the dual‐targeting biomimetic nanoparticles N_3_‐TIINPs were constructed. T cell membranes that were pretreated with Ac_4_GalNAz to label them with N_3_ groups via glycometabolism were extracted and then coated on prepared ICG‐PLGA polymeric cores by extrusion. Free ICG, ICG‐loaded PLGA‐lipid nanoparticles (INPs, using soybean lecithin instead of T cell membrane) and ICG‐loaded PLGA‐T cell membrane nanoparticles (TINPs) were used as controls. The typical core–shell structure of N_3_‐TINPs was visually characterized by transmission electron microscopy (TEM) and was found to have a size of 56 nm, which verified the successful fabrication of N_3_‐TINPs (**Figure** **2**A). The average diameter of PLGA core was 42 nm, and the surface area ratio of PLGA core and N_3_‐TINPs was 9:16. Due to the small size, N_3_‐TINPs with T cell natural properties could enter the tumor more easily. As measured by dynamic light scattering (DLS), the sizes of the INPs, TINPs, and N_3_‐TINPs were approximated (Figure 2B). The fluorescence (FL) spectra of the INPs, TINPs, and N_3_‐TINPs were consistent with the spectral profile of free ICG (Figure 2C). As shown in Figure S1 (Supporting Information), free ICG, INPs, TINPs, and N_3_‐TINPs showed similar absorption, with maximum absorption peak at 793 nm. According to the temperature curves and infrared thermal imaging (Figure 2D), the free ICG, INPs, TINPs, and N_3_‐TINPs exhibited alike temperature increase profiles, with maximum temperatures of 62, 67, 68, and 68 °C, respectively, under continuous 808 nm laser irradiation. By contrast, the maximum temperature of phosphate‐buffered saline (PBS) reached only 36 °C. This result indicated that the TINPs and N_3_‐TINPs showed the same photothermal conversion efficiency as the INPs and that the cell membrane coating had a negligible impact on the photothermal effects of the nano‐photosensitizer.

To confirm the successful preparation of N_3_‐TINPs, protein gel electrophoresis was used to analyze the protein composition on the T cell membranes of different groups. The protein profiles of N_3_‐TINPs and TINPs were close to those of T cell membrane vesicles and T cell lysate (Figure 2E), suggesting that T cell membrane proteins have been successfully retained on the prepared nanoparticles. Furthermore, Western blotting analysis was carried out to prove the presence of specific recognition molecules (e.g., TCRs) and N_3_ groups on the N_3_‐TINPs. As Figure 2F shows, the N_3_‐TINPs and TINPs maintained abundant specific recognition proteins (e.g., TCRs). Additionally, the N_3_‐TINPs coated with T cell membranes pretreated with Ac_4_GalNAz displayed abundant N_3_ groups. Cellular experiments further verified the successful generation of N_3_ groups on the surface of the target cells (Figure S2, Supporting Information). To confirm whether the N_3_ motif existed on the surface of N_3_‐TINPs, the N_3_‐TINPs was incubated with Raji cells, followed by stained with anti‐CD3‐FITC and DBCO‐Sata650, respectively. The confocal imaging showed CD3 membrane proteins and DBCO‐probe were nicely co‐located on Raji cells surface (Figure S3, Supporting Information), indicating N_3_ group still retain on the surface of N_3_‐TINPs.

### 2.2 Dual‐Targeting Property and Photothermal Cytotoxicity of N_3_‐TINPs

**Figure 3:**
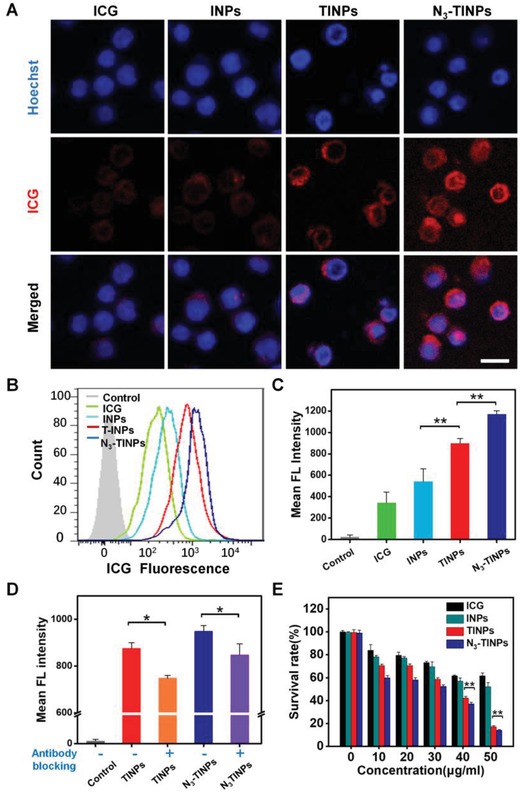
Validating the dual‐targeting property and photothermal cytotoxicity of N_3_‐TINPs in vitro. A) Evaluating the uptake of ICG in Raji cells by confocal microscopy. Scale bar: 25 µm. B) Flow analysis of Raji cells with different treatments. C) Quantification of cellular uptake in Raji cells. D) Inhibition of the recognition of Raji cells by TINPs and N_3_‐TINPs. TINPs and N_3_‐TINPs pretreated with anti‐TCR antibody, and cellular uptake in the Raji cells was analyzed using flow cytometry. E) Photothermal cytotoxicity analysis of N_3_‐TINPs at different concentrations. Statistical *P*‐values: (*) *P* < 0.05, (**) *P* < 0.01.

To investigate the immune recognition and bioorthogonal dual‐targeting ability of the N_3_‐TINPs, Raji tumor cells were pretreated with Ac_4_ManN‐BCN for 2 days to label them with the BCN group (Figure S4, Supporting Information). Ac_4_ManN‐BCN was previous reported by our group as a novel BCN modified unnatural sugar, which can efficient and nondestructive incorporated into wild tumor cell surface glycans, including human lung carcinoma cell line (A549 cells), human rhabdomyoma cell line (RD cells), human hepatocarcinoma cell line (HepG2 cells), and human breast cancer cell line (MCF‐7 cells).^12^ The confocal imaging exhibited that cells treated with N_3_‐TINPs showed obvious ICG fluorescence and that was higher than that observed in cells treated with TINPs, whereas cells treated with free ICG and INPs had very weak fluorescence (**Figure** **3**A). As expected, flow analysis displayed that compared to the cells treated with INPs, the fluorescence intensity of the Raji cells treated with TINPs yielded about onefold increase, owing to the immune recognition of the T cell membrane receptors. Furthermore, the fluorescence intensity of Raji cells treated with N_3_‐TINPs exceeded ≈34% compared to that treated with TINPs, due to the bioorthogonal reaction of the N_3_ group on the N_3_‐TINPs and the BCN group on the tumor cells. (Figure 3B,C). Simultaneously, after blocking TCR using anti‐TCR antibody,^14^ the cellular uptake of TINPs and N_3_‐TINPs was decreased, further confirming that the immune recognition of T cell membrane‐specific receptors plays an important role in targeting tumor cells (Figure 3D). The above results indicated that N_3_ group modification and the coating of the T cell membrane enhanced the efficiency of cellular uptake of N_3_‐TINPs due to their bioorthogonal targeting and immune recognition properties.

The in vitro photothermal toxicity of N_3_‐TINPs was further evaluated in Raji cells. Under the ICG concentration of 50 µg mL^−1^, the cell viabilities of Raji cells treated with TINPs and N_3_‐TINPs plus a laser were both less than 20%, whereas the viabilities of the cells in groups treated with free ICG or INPs were relatively high (62% and 53%, respectively). By contrast, the group treated with N_3_‐TINPs displayed the lowest cell viability and the most effective photothermal killing effect (Figure 3E). Simultaneously, flow analysis showed N_3_‐TINPs treatment induced more serious apoptosis in Raji cells under the same conditions when compared to TINPs, INPs, and ICG, which was consistent with the result of cell viability (Figure S5, Supporting Information).

### 2.3 In Vivo Biodistribution of N_3_‐TINPs

**Figure 4:**
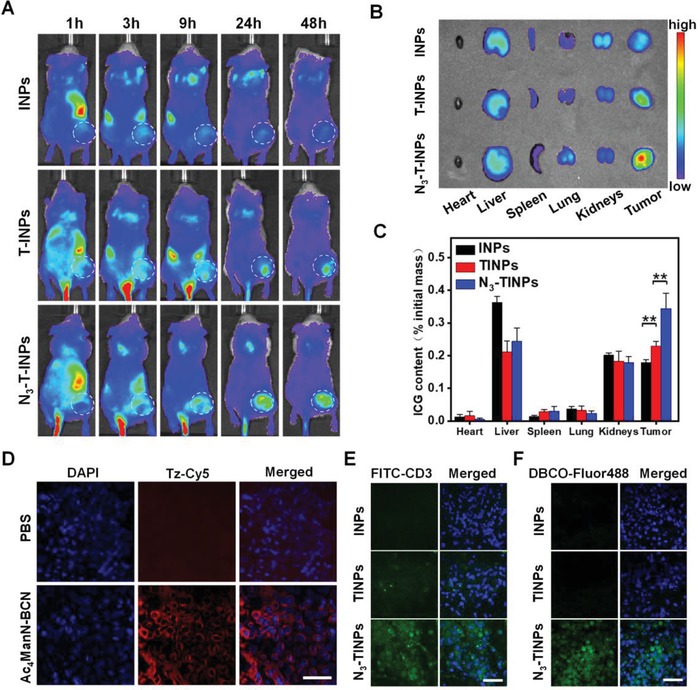
In vivo targeting and biodistribution of dual‐targeting N_3_‐TINPs. A) In vivo imaging analysis of mice after administered with different nanoparticles. B) The major organs and tumors of mice with the indicated treatment were harvested for ex vivo imaging at 48 h postinjection. C) The ICG content of each organ was quantified by fluorescence. D) The generation of BCN groups on tumor tissues were recorded by confocal microscopy. E,F) Evaluating the enrichment of N_3_‐TINPs in tumor tissues. The tumor tissue sections of mice administered different treatments were stained with anti‐CD3‐FITC and DBCO‐Flour 488. Scale bar: 50 µm. Statistical *P*‐values: (**) *P* < 0.01.

Considering the excellent targeting effect of N_3_‐TINPs in vitro, we further evaluated the targeted accumulation and biodistribution of N_3_‐TINP in the Raji cell‐bearing NOD/SCID mice. Tumor‐bearing mice were treated with or without Ac_4_ManN‐BCN by intratumoral administration,[qv: 11a,15] followed by intravenous (i.v.) injection of INPs, TINPs, or N_3_‐TINPs. **Figure** **4**A demonstrated that the INPs group exhibited a very weak fluorescence signal in tumor at 48 h after i.v. injection. By contrast, the TINPs and N_3_‐TINPs groups showed an obvious ICG fluorescence, suggesting that the T cell membrane coating clearly enhanced the accumulation of TINPs and N_3_‐TINPs in tumors. Significantly, the N_3_‐TINPs with immune recognition and bioorthogonal targeting capabilities showed the strongest fluorescence signal in tumor, indicated that dual‐targeting strategy has good specificity in vivo and remarkably improved the accumulation of N_3_‐TINPs. Furthermore, the heart, liver, spleen, lung, kidney, and tumors from sacrificed mice were harvested at 48 h post‐i.v. injection and taken for fluorescence measurement after homogenization. The N_3_‐TINPs treated group exhibited significant tumor accumulation, and the fluorescence intensity in the tumor was approximately onefold higher than that in groups treated with TINPs and INPs (Figure 4B,C). The above results suggest that N_3_‐TINPs possess outstanding dual‐targeting ability, and could more effectively access and accumulated in the tumor site.

The pharmacokinetics of INPs, TINPs and N_3_‐TINPs were investigated by measuring the ICG concentration in the blood at different time points after intravenous injection of nanoparticles to mice.^13^ As compared to INPs, ICG concentration in the blood of N_3_‐TINPs was significantly higher than that of INPs at 15 min post injection (Figure S6, Supporting Information). Moreover, the AUC_24 h_ (the area under the plasma drug concentration–time curve over the period of 24 h) of N_3_‐TINPs (360 µg min mL^−1^) was over fourfold higher than that of INPs (84 µg min mL^−1^), which would obviously enhance its bioavailability. To investigate the metabolic pathway of N_3_‐TINPs in mice, we measured the ICG content in urine and feces of mice at 24 h post‐injection. The excretion of urine ICG was an almost undetectable level, but fecal ICG excretion reached 14.59 ± 0.64 µg, suggesting that N_3_‐TINPs was metabolized from liver into intestine and subsequently into fecal excretion.^13^

Next, we investigated the generation of BCN groups on tumor tissues after intratumoral injection of Ac_4_ManN‐BCN. As Figure 4D reveals, after staining with tetrazine conjugated Cy5 (Tz‐Cy5, a probe for detecting BCN group), tumor tissues pretreated with Ac_4_ManN‐BCN showed conspicuous Cy5 fluorescence, while the control group without treatment had no obvious detectable fluorescence. These results indicated that BCN groups were successfully generated on the tumor cell surface. On this basis, we analyzed the accumulation of N_3_‐TINPs in tumor tissues. Confocal microscopy analysis of tumor tissues showed that various nanoparticles successfully entered the tumor tissues and that the N_3_‐TINPs group exhibited a distinct ICG fluorescence intensity (Figure S7, Supporting Information). Tumor tissues were stained with anti‐CD3‐FITC to detect the CD3 receptors on the T cell membrane or with dibenzocyclooctyne (DBCO) conjugated‐Fluor 488 to detect the N_3_ groups of N_3_‐TINPs. The results showed that tumors treated with N_3_‐TINPs displayed obvious FITC fluorescence and Fluor 488 fluorescence signal, while the tumors in the TINPs group showed only faint FITC fluorescence and the INPs group showed neither FITC nor Fluor 488 fluorescence (Figure 4E,F). The above results signified that the bioorthogonal reaction between BCN groups on tumor and N_3_ groups on N_3_‐TINPs was responsible for the outstanding accumulation of N_3_‐TINPs in tumors.

### 2.4 In Vivo Photothermal Therapeutic Efficacy and Biocompatibility of N_3_‐TINPs

**Figure 5:**
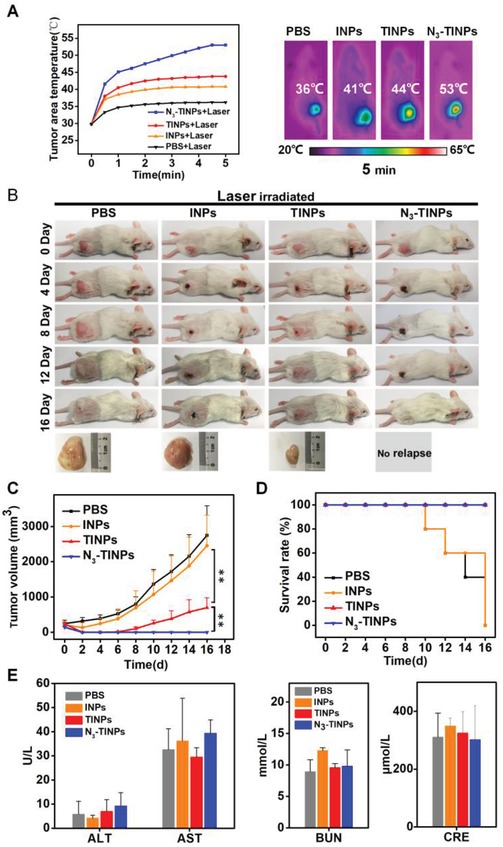
In vivo photothermal therapy efficacy and biocompatibility of dual‐targeting N_3_‐TINPs. A) Temperature curves and infrared thermal images of tumor‐bearing mice. B) Representative photos of mice bearing Raji tumors and excised tumors after 16‐day treatments. C) Raji tumor growth curves of different groups (*n* = 5). D) Survival rates of tumor‐bearing mice. E) Blood biochemistry analysis of liver and kidney function markers (ALT, AST, BUN, and CRE). Statistical *P*‐values: (*) *P* < 0.05, (**) *P* < 0.01.

We further evaluated the photothermal therapeutic efficacy of N_3_‐TINPs in mice. Tumor‐bearing mice pretreated as previous were randomly divided into four groups (*n* = 5 per group) and injected i.v. with PBS, INPs, TINPs, or N_3_‐TINPs, followed by laser irradiation (808 nm, 0.8 W cm^−2^ for 5 min) at 24 h after administration. As shown in **Figure** **5**A, the temperature of the tumor area in TINPs group increased to 44 °C, which exceed the damage threshold of tumor.[qv: 2a,16] Significantly, compared to the TINPs, the temperature in mice injected with N_3_‐TINPs rose to 53 °C, and the N_3_‐TINPs displayed an outstanding photothermal conversion ability. By contrast, the tumor temperature in mice treated with INPs and PBS rose to 41 and 36 °C, respectively, which were not enough to destroy the tumor. The infrared thermal images also visually exhibited the temperature of the tumors. The results demonstrated that N_3_‐TINPs have excellent photothermal effect due to their outstanding tumor‐targeted accumulation, indicating their effectiveness in photothermal ablation.

On this basis, the mice treated with TINPs plus laser displayed an obvious reduction in the tumor size compared to those in the PBS and INPs groups (Figure 5B,C); however, the treatment inhibited the tumor growth only partially in the first 6 days. Significantly, the N_3_‐TINPs group exhibited distinct tumor elimination with no relapse during the treatment, suggesting that N_3_‐TINPs have an evident photothermal effect attributable to their effective accumulation in tumors by dual targeting. The survival rates of mice treated with TINPs and N_3_‐TINPs were both 100%, whereas the mice treated with PBS and INPs all died after 16 days (Figure 5D). Additionally, we investigated proliferation level of tumor tissues after the administration of nanoparticles plus laser. The confocal images showed that Ki‐67 fluorescence signal could be clearly observed in control, while only weak fluorescence signals were detected in mimicking nanoparticle‐treated tumor tissues, especially in N_3_‐TINPs group (Figure S8, Supporting Information), indicating N_3_‐TINPs plus laser could inhibited tumor proliferation effectively.

Finally, we analyzed the biosafety of N_3_‐TINPs. The body weight of the mice did not obviously change after the treatment (Figure S9, Supporting Information). Additionally, the blood biochemical analysis indictors of mice receiving different treatments, including alanine aminotransferase (ALT), aspartate aminotransferase (AST), blood urea nitrogen (BUN), and creatinine (CRE), showed no significant difference, indicating that the side effects of our nanoparticles were negligible at the administered dose (Figure 5E).

## 3 Conclusion

In the present study, we successfully constructed a N_3_‐labeled T cell membrane nanoparticles (N_3_‐TINPs) platform with both bioorthogonal targeting and immune recognition properties. This platform highly increased the tumor targeting of nano‐photosensitizer, which greatly improved the effects of PTT. N_3_ and BCN groups can be artificially introduced to T cell or tumor cell surfaces with glycometabolic labeling respectively, in order to conduct bioorthogonal chemistry. The N_3_‐TINPs exhibited an outstanding photothermal response, an excellent fluorescence imaging properties and showed an excellent property to be anchored on tumor cells by a dual‐targeting mechanism. The accumulated N_3_‐TINPs in tumor improved the photothermal therapeutic efficacy and eradicate the tumor effectively without side effects. As a consequence, N_3_‐TINPs can serve as a promising drug delivery system with high‐targeting efficiency and therapeutic effect.

## 4 Experimental Section

*Materials*: Indocyanine green and poly (D, L‐lactide‐*co*‐glycolide) (PLGA, MW, 7000–17 000; lactide, glycolide (50:50)) were purchased from Sigma−Aldrich (USA). Soybean lecithin and 1,2‐distearoyl‐sn‐glycero‐3‐phosphoethanolamine‐*N*‐[carboxy (polyethylene glycol)‐2000] (DSPE‐PEG2000) were obtained from Avanti (USA). Hoechst 33 258 was purchased from Invitrogen (USA). Fetal bovine serum, penicillin−streptomycin, RPMI 1640 medium, AIM‐V medium, and human T‐Activator CD3/CD28 Dynabeads were acquired from Gibco Life Technologies (USA). Interleukin‐2 was purchased from Peprotech (USA). RIPA lysis buffer, Coomassie blue, and DAPI were provided by Beyotime Biotechnology (CHN). Mouse anti‐human T cell receptor *αβ*, and FITC conjugated anti‐CD3 were purchased from BD Biosciences (USA). Antibodies against Na^+^/K^+^‐ATPase were obtained from GenScript (USA). Horseradish peroxidase‐conjugated anti‐rabbit IgG and anti‐mouse IgG were acquired from Biolegend (USA). Dibenzocyclooctyne conjugated biotin (DBCO‐biotin), DBCO‐conjugated Fluor 488 (DBCO‐Fluor 488), N_3_‐conjugated fluorophore (N_3_‐Cy5.5), tetrazine‐conjugated fluorophore (Tz‐Cy5), and azido sugar Ac_4_GalNAz were obtained from Click Chemistry Tools (USA). Tetraacetylated‐*N*‐BCN‐carbonyl‐d‐mannosamine (Ac_4_ManN‐BCN) was offered by SIAT (CHN). Streptavidin‐horseradish peroxidase (streptavidin‐HRP) was purchased from Thermo Fisher Scientific. A 220 nm polycarbonate membrane and an Amicon ultra‐4 centrifugal filter with a molecular weight cutoff of 100 kDa were purchased from Merck Millipore (GER). Cell Counting Kit‐8 (CCK‐8) was purchased from Dojindo Laboratories (JPN).

*Activation and Glycometabolic Labeling of T Cells*: To obtain human T cells, human peripheral blood mononuclear cells (PBMCs) were stimulated by CD3/CD28 Dynabeads and cultivated in AIM‐V medium containing 2% fetal bovine serum and interleukin‐2 at 37 °C with 5% CO_2_ in a humidified incubator. Azido sugar Ac_4_GalNAz (50 × 10^−6^ m) was added to T cells and incubated for 2 days, then acquired the activated azide (N_3_)‐labeling T cells. In the experiment of acquisition of PBMCs, healthy adults were recruited and blood was drawn from the arm vein. The experiment was carried out by experienced nurses in the formal Shenzhen Hospital with protective measures and does not involve special privacy. The corresponding human experiments conform to the ethics of the Institutional Review Board of SIAT (serial number: SIAT‐IRB‐170315‐H0145).

*T Cell Membrane Extraction*: The extrusion approach reported previously was used.^13^ With hypotonic lysis, mechanical membrane disruption (VCX130 ultrasonics processor, USA) and then differential‐speed centrifugation (OptimaTM MAX‐XP, Beckman, USA), the T cell membrane (10^7^) was obtained.

*Synthesis of N_3_‐TINPs*: 1 mg ICG was dissolved in ethanol solution (4%), and then dropwise added 0.5 mL of PLGA solution (dissolved in acetonitrile, 4 mg mL^−1^) under sonication at a power of 39 W and a frequency of 20 kHz for 5 min to form an ICG‐PLGA core. The membrane material was mixed with 90 µg of DSPE‐PEG2000 and physically extruded by a 220 nm polycarbonate membrane, resulting N_3_‐T cell membrane vesicles. The N_3_‐TINPs were obtained by coextruding the vesicles and cores through a 220 nm polycarbonate membrane. The TINPs and INPs were obtained by the same method as the N_3_‐TINPs except that T cell membrane and soybean lecithin, respectively, were substituted for azide‐T cell membrane.

*Characterization of N_3_‐TINPs*: The size distribution of the nanoparticles was obtained by dynamic light scattering (Malvern Zetasizer Nano ZS90). The nanoparticle morphology and structure were acquired with a transmission electron microscope (Tecnai G2 F20 S‐Twin, USA). The ultraviolet absorption spectra and the fluorescence spectra (with an emission wavelength of 740 nm) were measured by UV–vis spectrometry (Lambda25, Perkin−Elmer, USA) and fluorescence spectroscopy (F900, Edinburgh Instruments Ltd., UK) respectively. Quartz cuvettes with the N_3_‐TINPs, TINPs, INPs, free ICG, and PBS were irradiated with 808 nm laser (Leimai, CHN) for 10 min (0.8 W cm^−2^). Using an infrared thermal imaging camera (Fluke Ti27, USA) to acquire infrared thermographic maps.

The membrane proteins and N_3_‐glycoproteinon of the N_3_‐TINPs were characterized by sodium dodecyl sulfate‐polyacrylamide gel electrophoresis (SDS‐PAGE). The T cells, T cell membranes vesicles, TINPs, and N_3_‐TINPs were lysed with RIPA lysis buffer and the membrane proteins were extracted, then measured by the BCA Assay Kit (Beyotime, CHN). All samples were mixed with SDS‐PAGE sample loading buffer and heated at 100 °C for 10 min. Samples were added into the wells of 10% SDS‐PAGE gel equivalently for electrophoresis analysis in a Mini‐PROTEAN Tetra System (Bio‐Rad, USA). Protein was stained by Coomassie blue and destained in ddH_2_O overnight before imaging.

For Western blot analysis, the protein was transferred to a polyvinylidene fluoride (PVDF) membrane using the Mini‐PROTRAN Tetra System (Bio‐Rad, USA) at 200 mA for 2 h. The membranes were detected using anti‐TCR, and anti‐Na^+^/K^+^‐ATPase along with either horseradish peroxidase‐conjugated anti‐rabbit IgG or anti‐mouse IgG. DBCO‐biotin and streptavidin‐HRP were used to probe the N_3_ groups on the membrane. Finally, the enhanced chemiluminescence method was adopted to measure the protein signals using a ChemiDoc MP gel imaging system (Bio‐Rad, USA). To further confirm whether the N_3_ motif existed on the surface of N_3_‐TINPs particles, Raji cells were incubated with 20 µg mL^−1^ of INPs, TINPs or N_3_‐TINPs for 1 h, and then stained with anti‐CD3‐FITC and DBCO‐Sata650, respectively. Confocal fluorescent images were obtained by a laser confocal scanning microscope (Leica, GER).

*Tumor Cell Culture and Glycometabolic Labeling*: Raji tumor cells were cultured in RPMI 1640 medium containing 10% fetal bovine serum and 1% penicillin‐streptomycin at 37 °C in a humidified atmosphere of 5% CO_2_. Bicyclo [6.1.0] nonyne modified sugar Ac_4_ManN‐BCN (40 × 10^−6^ m) was added to Raji cells and incubated for 2 days to decorate the BCN group on tumor cells.

*Determination of the Generation of Azide and BCN Groups on the Cell Surface*: Raji cells pretreated with or without Ac_4_ManN‐BCN (40 × 10^−6^ m) for 2 days were incubated with N_3_‐Cy5.5, and the fluorescence intensity was obtained by confocal laser scanning microscopy (CLSM) imaging and flow cytometric analysis. T cells pretreated with or without Ac_4_GalNAz (50 × 10^−6^ m) for 2 days were stained with DBCO‐Fluor 488 and detected by the same method.

*In Vitro Cellular Uptake and Targeting Validation of N_3_‐TINPs*: CLSM and flow cytometry were used to analyze the uptake of Raji cells. After pretreatment with Ac_4_ManN‐BCN, Raji cells were incubated with N_3_‐TINPs, TINPs, INPs, or free ICG (20 µg mL^−1^ of ICG) for 45 min at 4 °C. The cells were washed three times with PBS, and the fluorescence of ICG was detected by a flow cytometer (BD Accuri C6, USA). The Raji cells (1 × 10^5^ cells/well) were seeded in eight‐well chambered coverglasses (Lab‐Tek, Nunc, USA). The fresh medium containing N_3_‐TINPs, TINPs, INPs, or free ICG (20 µg mL^−1^ of ICG) was added. After 45 min of incubation, the cells were washed three times with PBS, then stained with Hoechst 33 258 for 5 min and rinsed with PBS. Cellular fluorescence was observed with a TCS SP5 confocal laser scanning microscope (Leica, GER).

In the blocking experiment,^14^ the recognition of TCRs on the T cell membrane by tumors was inhibited using anti‐TCR antibody. TINPs and N_3_‐TINPs were pretreated with anti‐TCR (2.5 µg mL^−1^) for 45 min at 4 °C, followed by incubation with Raji cells under the same conditions. The cellular uptake of TINPs and N_3_‐TINPs in Raji cells was analyzed by flow cytometry.

*In Vitro Photothermal Toxicity of N_3_‐TNPs*: Raji cells (1 × 10^5^ cells/well) were seeded in a 96‐well plate in medium (100 µL) containing N_3_‐TINPs, TINPs, INPs, or free ICG. After 45 min of incubation at 4 °C, the cells were washed with PBS, and resuspended with fresh medium. The cell viability was measured via a CCK‐8 assay after irradiated with an 808 nm laser (0.8 W cm^−2^, 5 min). Simultaneously, the apoptosis of Raji cells with the same treatments was measured and analyzed using Apoptosis Detection Kits (BD) as the manufacturer's protocols.

*Animal Model*: Female NOD/SCID mice (6–8 weeks old) were purchased from Vital River Animal Technology Co. Ltd. The protocols were approved by the Animal Care and Use Committee (Shenzhen Institutes of Advanced Technology, Chinese Academy of Sciences). The Raji tumors were generated by subcutaneous injection of 1 × 10^7^ cells in the flank region.

*In Vivo Imaging and Biodistribution Analysis*: When the tumors reached an average volume of 100–200 mm^3^, the NOD/SCID mice were injected with N_3_‐TINPs, TINPs, INPs (150 µL, 400 µg mL^−1^ ICG), or PBS via the tail vein. The fluorescence signals of ICG were measured with an IVIS Spectrum imaging system (Maestro, USA). The mice were euthanized after 48 h, and the major organs (heart, liver, spleen, lung, kidneys) and tumors were excised and detected by the IVIS Spectrum image system (Maestro, USA).

The content of ICG in major organs and tumors were quantitatively detected by FL spectrometry. The major organs, tumors and urine/feces were homogenized in 6 mL of dimethyl sulfoxide (DMSO) to extract the ICG, and then centrifuged for 15 min at 9000 rpm.^13^ Blood samples of mice were centrifuged at 16 000 g for 5 min, and then the plasma was used to evaluate the blood circulation time curve.^13^ The ICG content of each sample was determined by FL spectrometry.

*Immune Fluorescence Analysis of Tumor Tissues*: The tumor tissues were excised from tumor‐bearing mice 24 h after administration, cut into 6 µm sections, and then fixed in 4% formaldehyde solution. The tumor tissues were incubated for 10 min in PBS containing 0.25% Triton X‐100 for permeabilization. Then, the cells were incubated with 1% BSA for 30 min to block nonspecific binding of the antibodies. Tumor tissues were stained with anti‐CD3‐FITC antibody for 2 h or DBCO‐Fluor 488 for 30 min at room temperature and then washed with PBS. Additionally, other tumor tissues were stained with Tz‐Cy5 for 30 min at room temperature and washed with PBS. The tumor tissues were both stained with DAPI for 3–5 min and washed with PBS. A confocal laser scanning microscope (Leica, GER) was used to detect the fluorescence in the tumor tissues. To analysis the tumor proliferation after photothermal therapy, the above tumor tissues with laser irradiation were excised from mice, and then the confocal immunofluorescent analysis were performed using anti‐Ki‐67 antibodies (CST) as the standard protocol.

*In Vivo Antitumor Efficacy and Biosafety of N_3_‐TINPs*: Tumor‐bearing NOD/SCID mice were intravenous (i.v.) injected with N_3_‐TINPs, TINPs, INPs (250 µL, 350 µg mL^−1^ ICG), or PBS (250 µL). At 24 h after injection, the tumors were irradiated with a laser (808 nm, 0.8 W cm^−2^) for 5 min. The maximum temperatures of the tumors and infrared thermographic maps were acquired by a Ti27 infrared thermal imaging camera (Fluke, USA) during the laser irradiation. The body weight of the mice and volumes of the tumors were recorded every 2 days during the period of treatment (Tumor volume = Length × Width^2^ / 2). Mice with tumor sizes over 1500 m^3^ were assumed to be dead. The blood biochemistry indicators, including alanine aminotransferase, aspartate aminotransferase, blood urea nitrogen, and creatinine, were evaluated according to the manufacturer's instructions (JianCheng Biotech, CHN).

*Statistical Analysis*: All the results are reported as mean ± SD. The differences among groups were analyzed using one‐way ANOVA analysis and Student's *t*‐test; (*) *P* < 0.05, (**) *P* < 0.01.

## Conflict of Interest

The authors declare no conflict of interest.

## Supporting information

Supplementary
