# Supplementary material for: T Cell Membrane Mimicking Nanoparticles with Bioorthogonal Targeting and Immune Recognition for Enhanced Photothermal Therapy
Source: Adv Sci (Weinh). 2019 Jun 11;6(15):1900251. doi: 10.1002/advs.201900251 (PMC6685477; doi:10.1002/advs.201900251)
Supplement: Supplementary file 1 — Supplementary [file ADVS-6-1900251-s001.pdf]

Copyright WILEY-VCH Verlag GmbH & Co. KGaA, 69469 Weinheim, Germany, 2019.

ADVANCED SCIENCE Open Access

Supporting Information

for *Adv. Sci.,* DOI: 10.1002/advs.201900251

T Cell Membrane Mimicking Nanoparticles with Bioorthogonal Targeting and Immune Recognition for Enhanced Photothermal Therapy

Yutong Han, Hong Pan, Wenjun Li, Ze Chen, Aiqing Ma, Ting Yin, Ruijing Liang, Fuming Chen, Yifan Ma, Yan Jin, Mingbin Zheng, Baohong Li,* and Lintao Cai**

Supporting Information

Title: T Cell Membrane Mimicking Nanoparticles with Bioorthogonal Targeting and Immune Recognition for Enhanced Photothermal Therapy

Yutong Han, Hong Pan, Wenjun Li, Ze Chen, Aiqing Ma, Ting Yin, Ruijing Liang, Fuming Chen, Yifan Ma, Yan Jin, Mingbin Zheng, Baohong Li*, and Lintao Cai**

Yutong Han, Hong Pan, Wenjun Li, Ze Chen, Ting Yin, Ruijing Liang, Fuming Chen, Yifan Ma, Yan Jin, Mingbin Zheng, and Lintao Cai
Guangdong Key Laboratory of Nanomedicine, CAS-HK Joint Lab of Biomaterials, Shenzhen Engineering Laboratory of Nanomedicine and Nanoformulations, Shenzhen Institutes of Advanced Technology (SIAT), Chinese Academy of Sciences, Shenzhen 518055, P. R. China
E-mail: L. Cai (lt.cai@siat.ac.cn), B. Li (gdmcli@126.com), and M. Zheng (mb.zheng@siat.ac.cn)

Yutong Han, Aiqing Ma, Fuming Chen, Baohong Li, and Mingbin Zheng
Dongguan Key Laboratory of Drug Design and Formulation Technology, Key Laboratory for Nanomedicine, Guangdong Medical University, Dongguan 523808, PR China

Supplementary Figures

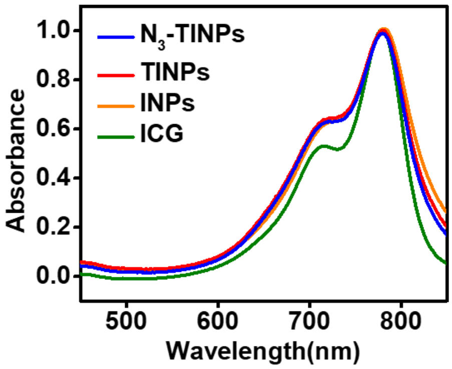

Figure S1. UV-vis absorption spectra of N_3-TINPs, TINPs, INPs and free ICG.

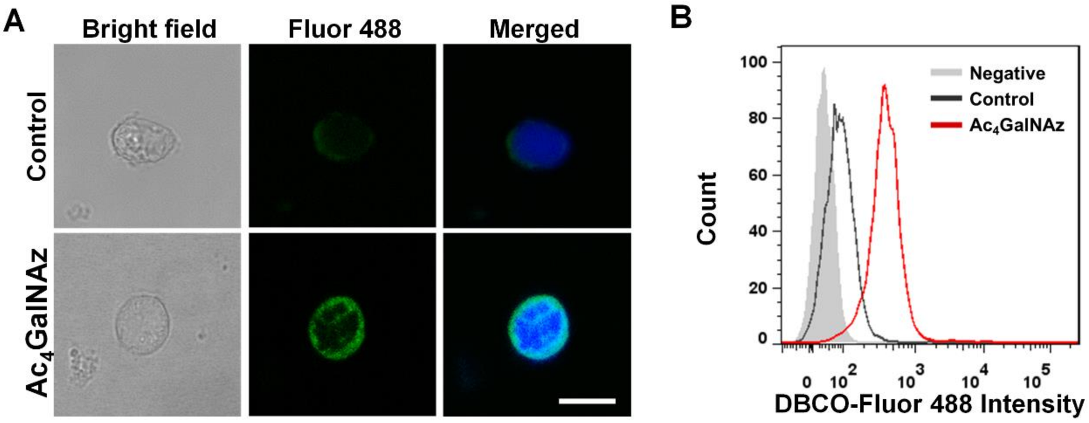

Figure S2. Verification of the generation of azide groups on the T cell surface after pretreatment with the $Ac_4GalNAz$. (A) CLSM images of T cells pretreated with $Ac_4GalNAz$ and then reacted with DBCO-Fluor 488. (B) Flow cytometric analysis of T cells after incubation with $Ac_4GalNAz$ followed by reaction with DBCO-Fluor 488. Scale bar: 10 μm.

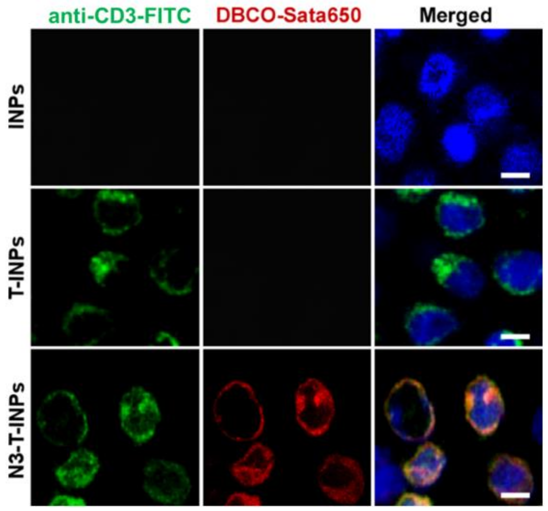

Figure S3. N_3 motif identification on the surface of N_3-TINPs . Raji cells were incubated with 20 μg mL^{-1} of INPs, TINPs or N_3-TINPs for 1 h, and then stained with anti-CD3-FITC and DBCO-Sata650, respectively. Fluorescent images were obtained and analyzed by a laser confocal scanning microscope. Scale bar: 10 μm.

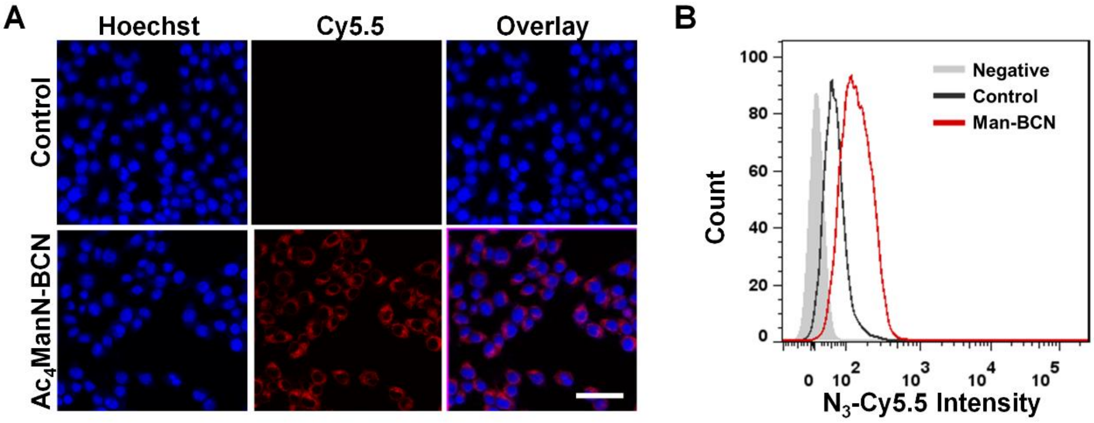

Figure S4. Verification of the generation of BCN groups on the Raji cell surface after pretreatment with the Ac_4ManN-BCN. (A) CLSM images of Raji cells pretreated with Ac_4ManN-BCN and then reacted with N_3- Cy5.5. (B) Flow cytometric analysis of Raji cells after incubation with Ac_4ManN-BCN followed by reaction with N_3-Cy5.5. Scale bar: 50 μm.

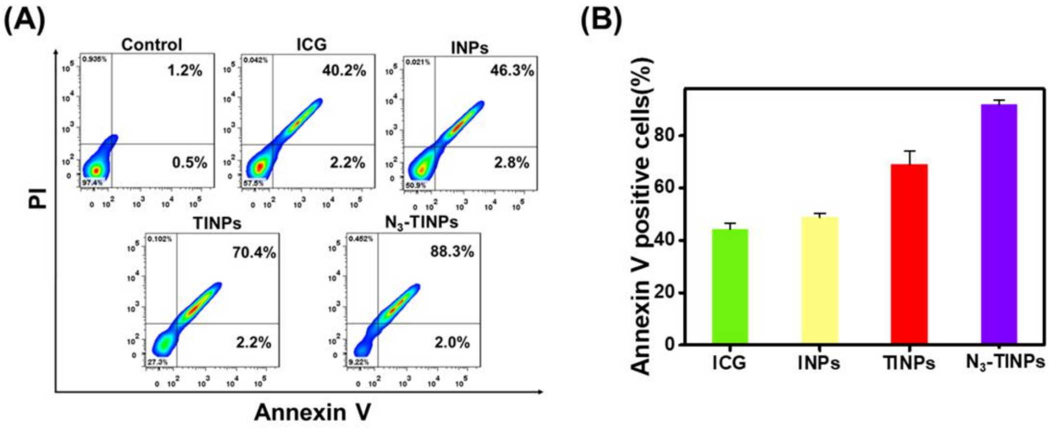

Figure S5. The apoptosis of Raji cells after laser irradiation. (A) Flow cytometry analysis of Raji cells apoptosis. (B) Apoptosis rate quantification of Raji cells.

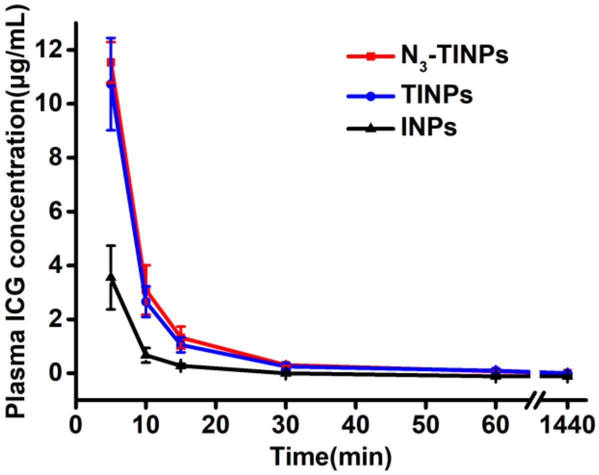

Figure S6. The pharmacokinetic parameters of N_3-TINPs *in vivo*. Healthy mice (6 weeks old) were intravenously injected with INPs, TINPs and N_3-TINPs (250 μL, 350 μg mL^{-1} ICG). Blood samples were collected at 5 min, 10 min, 15 min, 30 min, 1 h and 24 h (n = 3 at each time point), and the plasma ICG concentrations at the indicated time points were analyzed by fluorescence quantitation.

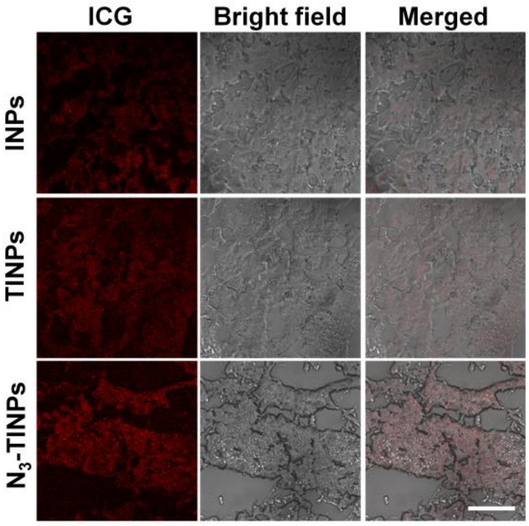

Figure S7. CLSM imaging of tumor tissue sections from mice after administration of different nanoparticles. Scale bar: 50 μm.

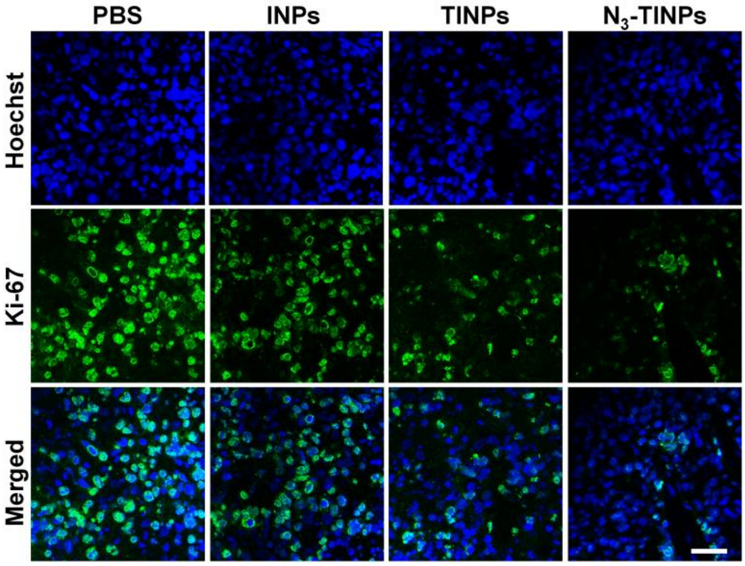

Figure S8. Tumor proliferation after photothermal therapy of N_3-TINPs . After laser irradiation, the tumor tissues were excised from mice, and then the confocal immunofluorescent analysis were preformed using anti-Ki-67 antibodies as the standard protocol. The fluorescent signal of Ki-67 protein indicate the proliferation level of tumor cells. Scale bar: 50 μm.

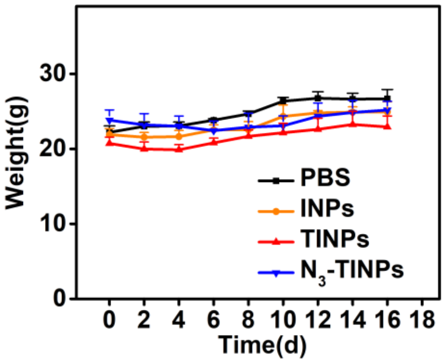

Figure S9. Body weights were measured during the 16-day evaluation period in mice under the different conditions.
